# Glucuronomannan GM2 from *Saccharina japonica* Enhanced Mitochondrial Function and Autophagy in a Parkinson’s Model

**DOI:** 10.3390/md19020058

**Published:** 2021-01-25

**Authors:** Yingjuan Liu, Weihua Jin, Zhenzhen Deng, Quanbin Zhang, Jing Wang

**Affiliations:** 1 Key Laboratory of Experimental Marine Biology, Center for Ocean Mega-Science, Institute of Oceanology, Chinese Academy of Sciences, Qingdao 266071, China; liuyjmiao@163.com (Y.L.); dengzhenzhen16@mails.ucas.ac.cn (Z.D.); qbzhang@qdio.ac.cn (Q.Z.); 2 School of Basic Medicine, Qingdao University, Qingdao 266071, China; 3 Laboratory for Marine Biology and Biotechnology, Qingdao National Laboratory for Marine Science and Technology, Qingdao 266071, China; 4 College of Biotechnology and Bioengineering, Zhejiang University of Technology, Hangzhou 310014, China; jinweihua@zjut.edu.cn

**Keywords:** glucuronomannan oligosaccharide, Parkinson’s disease, apoptosis, autophagy, mitochondria

## Abstract

Parkinson’s disease (PD), one of the most common neurodegenerative disorders, is caused by dopamine depletion in the striatum and dopaminergic neuron degeneration in the substantia nigra. In our previous study, we hydrolyzed the fucoidan from *Saccharina japonica*, obtaining three glucuronomannan oligosaccharides (GMn; GM1, GM2, and GM3) and found that GMn ameliorated behavioral deficits in Parkinsonism mice and downregulated the apoptotic signaling pathway, especially with GM2 showing a more effective role in neuroprotection. However, the neuroprotective mechanism is unclear. Therefore, in this study, we aimed to assess the neuroprotective effects of GM2 in vivo and in vitro. We applied GM2 in 1-methyl-4-phenylpyridinium (MPP^+^)-treated PC12 cells, and the results showed that GM2 markedly improved the cell viability and mitochondrial membrane potential, inhibited MPP^+^-induced apoptosis, and enhanced autophagy. Furthermore, GM2 contributed to reducing the loss of dopaminergic neurons in 1-Methyl-4-phenyl-1,2,3,6-tetrahydropyridine (MPTP)-induced mice through enhancing autophagy. These data indicate that a possible protection of mitochondria and upregulation of autophagy might underlie the observed neuroprotective effects, suggesting that GM2 has potential as a promising multifunctional lead disease-modifying therapy for PD. These findings might pave the way for additional treatment strategies utilizing carbohydrate drugs in PD.

## 1. Introduction

Parkinson’s disease (PD), a chronic neurodegenerative disease named after James Parkinson, is caused by dopamine depletion in the striatum and dopaminergic neuron degeneration in the substantia nigra [1,2,3]. The typical clinical symptoms of PD comprise progressive motor deficits, including bradykinesia, muscle tone rigidity, resting tremor, and postural instability [4]. In addition, PD patients display several nonmotor symptoms, such as sleep disorders, dementia, and sensory abnormalities [5]. Dopamine replacement therapy is a common treatment for PD currently, but it may produce many adverse effects and does not alter the progression of PD. For the most part, environmental toxins and genetic predisposition are considered to contribute to the disease onset [6]. Oxidative stress, neuroinflammation, mitochondrial malfunction, and autophagy inhibition are recognized as important contributors to PD pathogenesis [7,8,9]. In particular, misfolding and intracellular aggregation of α-synuclein into Lewy bodies are thought to be crucial steps in the pathogenesis of PD [10].

Increasing evidence has shown that an impairment of autophagy plays a fundamental role in the development and progression of PD [11]. Autophagy is a cellular pathway involved in balancing energy sources, in degrading damaged organelles and misfolded proteins, and in acting as an intracellular survival mechanism connected to human disease and physiology [12,13]. Phosphorylation and sumoylation have both been reported to accelerate α-synuclein turnover through macroautophagy [14]. Therefore, the upregulation of autophagy may represent a new therapeutic strategy for PD. 

In recent years, natural products have been proposed for the prevention and/or delay in the development of neurodegenerative disorders like PD [15,16,17,18]. Among them, polysaccharides are receiving attention due to their diverse and specific chemical compositions [19,20,21]. Our previous study found that the fucoidan from *Saccharina japonica* exhibited neuroprotective effects in H_2_O_2_-induced SH-SY5Y cells through the inhibition of apoptosis [22]. Considering its high molecular weight and complex structure, we hydrolyzed the polysaccharide, obtaining three glucuronomannan oligosaccharides (GMn; GM1, GM2, GM3) and applied the oligosaccharides in a MPTP-induced Parkinson’s model. The results revealed that GMn ameliorated behavioral deficits in Parkinsonism mice and downregulated the apoptotic signaling pathway, especially with GM2 showing a more effective role in neuroprotection [23]. However, how did GM2 play the neuroprotective role? It is reported that fucoidan may act as a biopolymer to induce autophagy [24]. GM2 is the backbone of fucoidan; therefore, we speculated that GM2 might exert its neuroprotective role by regulating autophagy. In this study, we further detect the neuroprotective effects of GM2 in vivo and in vitro.

## 2. Results

### 2.1. GM2 Improves Cell Viability and Suppresses Apoptosis in PC12 Cells

**Figure 1:**
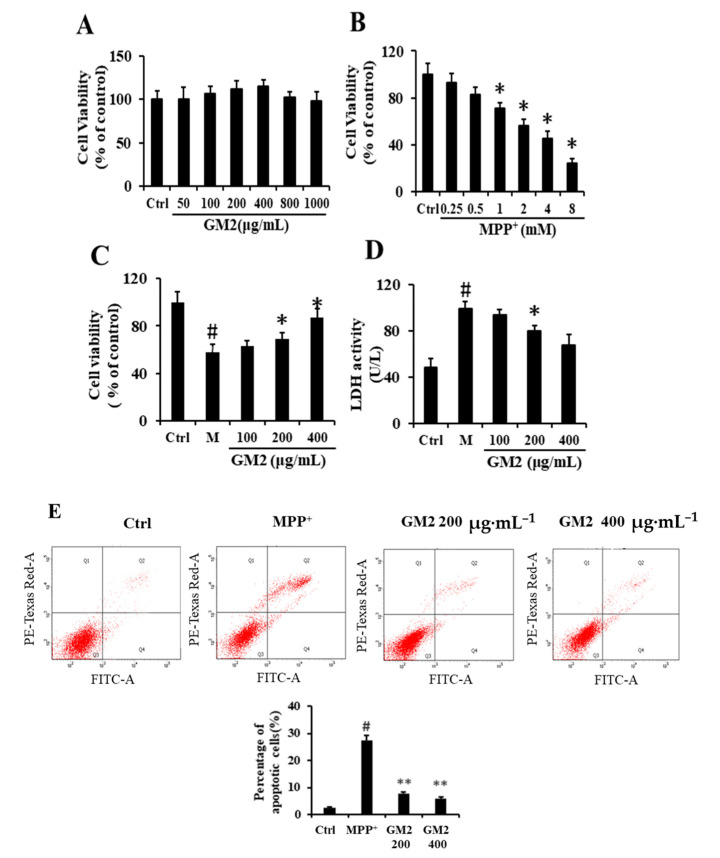
Glucuronomannan oligosaccharide 2 (GM2) improved cell viability and suppressed apoptosis in PC12 cells: (**A**,**B**) cell viability of PC12 cells treated with GM2 or 1-methyl-4-phenylpyridinium (MPP^+^) for 24 h, (**C**) cell viability, (**D**) LDH release, and (**E**) annexin V-fluorescein isothiocyanate (FITC)/propidium iodide (PI) staining from PC12 cells treated with GM2 for 12 h followed by MPP^+^ for 24 h. The values are given as mean ± SEM (*n* = 5). ^#^ *p* < 0.05 versus the control cells; * *p* < 0.05 versus the cells treated with MPP^+^ alone. ** *p* < 0.05 versus the cells treated with MPP^+^ alone. GM2: 200 and 400 µg·mL^−1^; MPP^+^: 2 mmol·L^−1^.

MPP^+^ (1-methyl-4-phenylpyridinium) is a neurotoxin that is transformed from MPTP by the enzyme monoamine oxidase-B (MAO-B) in astrocytes [25]. To evaluate the potential protective effects of GM2, we applied GM2 in MPP^+^-induced PC12 cells. The results showed that GM2 treatment did not alter cell viability (Figure 1A); however, MPP^+^ treatment decreased cell viability in a dose-dependent manner; 2 mmol·L^−1^ of MPP^+^ reduced cell viability to 55.2% ± 1.38 (Figure 1B), so this concentration was applied for subsequent experiments. After treatment with GM2 (200 or 400 µg·mL^−1^), cell viability was significantly increased (Figure 1C). These results were supported by lactate dehydrogenase (LDH) leakage, showing that GM2 (400 µg·mL^−1^) inhibited LDH release (Figure 1D). LDH, as an important cytosolic enzyme, is an indication of toxicant-induced cell death [26]. These observations indicated that GM2 possesses potent neuroprotective effects in dopaminergic neurons. To further estimate the protective effects of GM2 against MPP^+^, we applied the annexin V-fluorescein isothiocyanate (FITC)/propidium iodide (PI) assay (Figure 1E). The percentage of apoptotic cells in the MPTP group was significantly more than that in the control group, with a statistically significant difference, while administration of GM2 markedly inhibited the trend.

### 2.2. GM2 Ameliorates MMP and Improves Antioxidant Capacity in PC12 Cells

**Figure 2:**
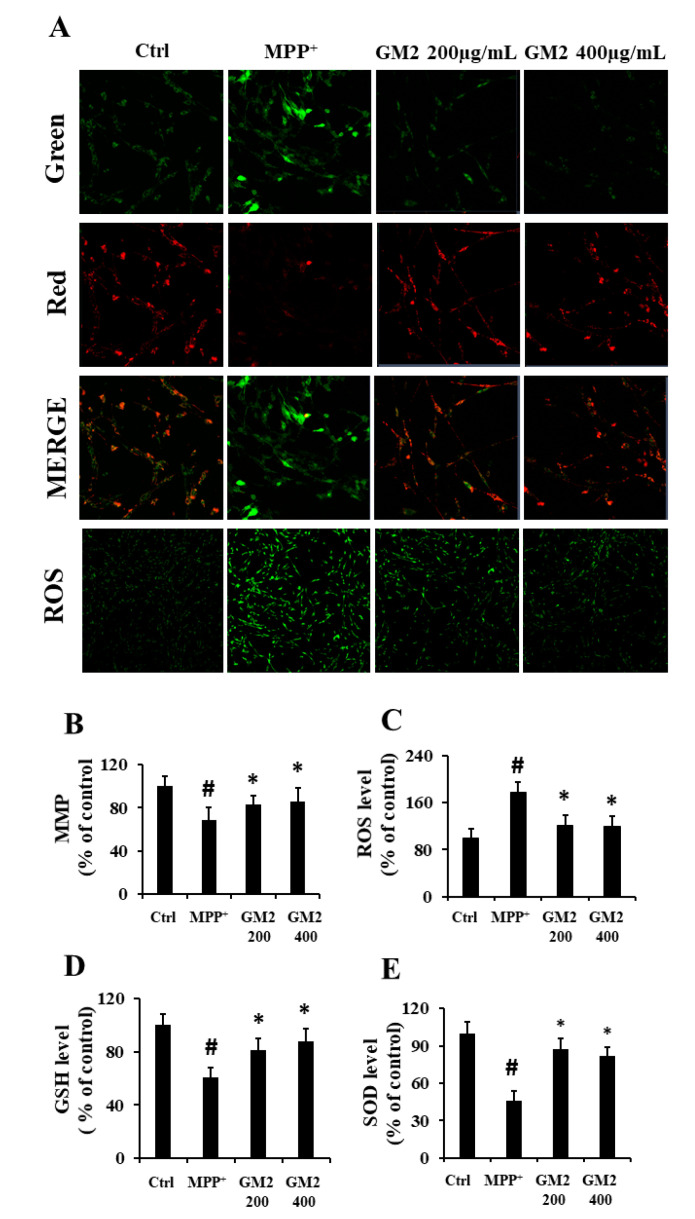
GM2 ameliorated the mitochondrial membrane potential (MMP) and improved the antioxidant capacity in PC12 cells. PC12 cells were treated with GM2 for 12 h followed by MPP^+^ for 24 h: (**A**) JC-1 staining (scale bar = 50 μm) and dichlorofluorescein diacetate (DCFH-DA) staining (scale bar = 200 μm), (**B**) the ratio of MMP, (**C**) the ratio of ROS, (**D**) GSH level, and (**E**) SOD level. The values are given as mean ± SEM (*n* = 3). ^#^ *p* < 0.05 versus the control cells; * *p* < 0.05 versus the cells treated with MPP^+^ alone. GM2: 200 and 400 µg·mL^−1^; MPP^+^: 2 mmol·L^−1^.

Mitochondrial membrane potential (MMP), is the most direct and simplest measurement reflecting the function of mitochondria [27]. In this study, we applied mitochondrial membrane potential (JC-1) to detect changes in the MMP of cells. As shown in Figure 2, after exposure to MPP^+^, the mitochondrial membrane potential decreased (Figure 2A,B), implying that MPP^+^ damaged the integrity of the mitochondrial membrane. However, GM2 significantly attenuated this injury. Mitochondria is one of the major sources of reactive oxygen species (ROS) and, in return, is highly susceptible to oxidative damage [28,29]. ROS levels were detected next using dichlorofluorescein diacetate (DCFH-DA). The green fluorescence intensity is proportional to the ROS levels. As shown in Figure 2C, the production of ROS in the MPP^+^ group significantly increased compared to the control group while GM2 remarkably reduced the spread of ROS, indicating that GM2 effectively antagonizes MPP^+^-induced increases in ROS levels, which might be caused by oxidative stress. The overproduction of ROS and superoxide play an important role in MPP^+^-inflicted oxidative damage and cytotoxicity [30]. Therefore, we next evaluated the effect of GM2 on MPP^+^-induced oxidative stress. The treatment of MPP^+^ markedly inhibited the levels of glutathione (GSH) and suppressed the activity of superoxide dismutase SOD in PC12 cells, while administration of GM2 attenuated the loss of GSH and SOD (Figure 2D,E), indicating that GM2 facilitates the antioxidative system to protect mitochondria from oxidative stress.

### 2.3. GM2 Inhibits Apoptosis in PC12 Cells

**Figure 3:**
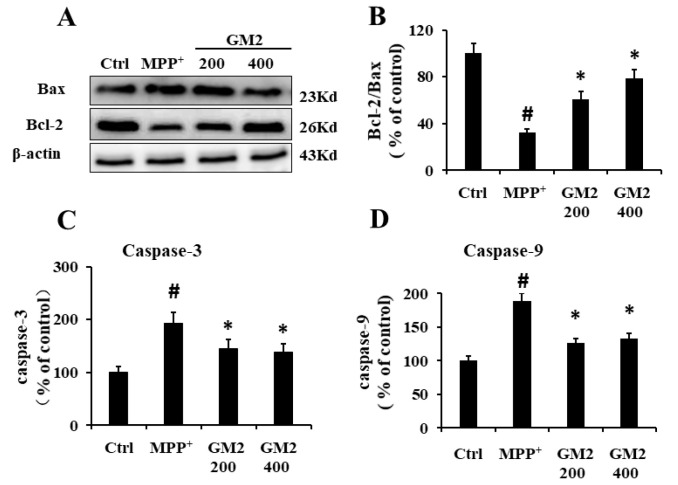
GM2 inhibited apoptosis in PC12 cells. PC12 cells were treated with GM2 for 12 h followed by MPP^+^ for 24 h: (**A**) the original bands of Bcl-2 and Bax, (**B**) the ratio of Bcl-2/Bax, and the relative activities of caspase-3 (**C**) and caspase-9 (**D**). The values are given as mean ± SEM (*n* = 3). ^#^ *p* < 0.05 versus the control cells; * *p* < 0.05 versus the cells treated with MPP^+^ alone. GM2: 200 and 400 µg·mL^−^^1^; MPP^+^: 2 mmol·L^−1^. Scale bar = 200 μm.

It has been demonstrated that Bcl-2 and Bax are vital members of the Bcl-2 family proteins and that the ratio of Bcl-2/Bax determines the fate of cells [31,32]. In our study, we found that treatment with GM2 improved cell viability, so we speculated that GM2 might inhibit apoptosis in PC12 cells. As shown in Figure 3A,B, MPP^+^ improved the expression of proapoptotic Bax and inhibited expression of antiapoptotic Bcl-2 while GM2 enhanced the ratio of Bcl-2/Bax, attenuating apoptosis. A similar result was observed in caspase-9 in which GM2 significantly restrained the activation of caspase-9 induced by MPP^+^ (Figure 3D). Of note, caspase-3, an eventual protein in the apoptotic cascade that catalyzed the specific cleavage of multiple vital cellular proteins [33], was also reduced in response to the administration of GM2 in PC12 cells (Figure 3C), indicating that GM2 inhibits apoptosis in MPP^+^-induced PC12 cells.

### 2.4. GM2 Enhances Autophagy in PC12 Cells

**Figure 4:**
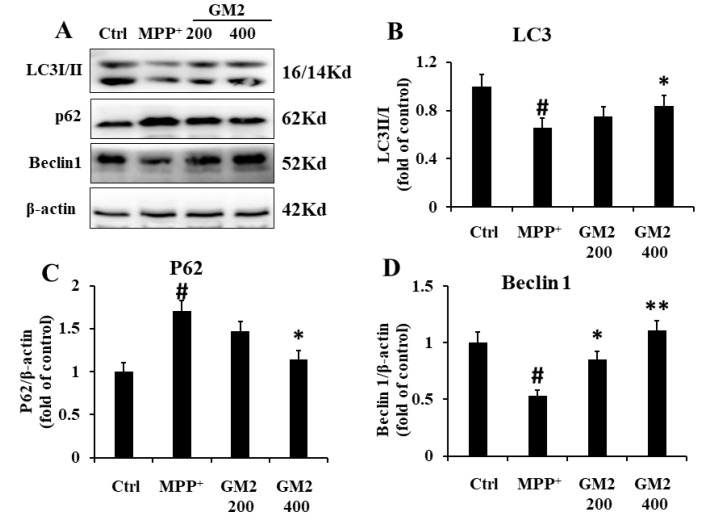
GM2 enhanced autophagy in PC12 cells. PC12 cells were treated with GM2 for 12 h followed by MPP^+^ for 24 h: (**A**) the original bands of LC3B, P62, and Beclin1; (**B**) the ratio of LC3 II/I; (**C**) the ratio of P62; and (**D**) the ratio of Beclin1. The values are given as mean ± SEM (*n* = 3). ^#^ *p* < 0.05 versus the control cells; * *p* < 0.05 and ** *p* < 0.05 versus the cells treated with MPP^+^ alone. GM2: 200 and 400 µg·mL^−1^; MPP^+^: 2 mmol·L^−1^. Scale bar = 200 μm.

As shown in Figure 4, we observed MPP^+^-attenuated levels of Beclin1 and LC3 II and increased levels of p62, suggesting that a block in autophagy occurred in PC12 cells. However, GM2 treatment greatly enhanced autophagy. Collectively, treatment with GM2 demonstrated beneficial effects in PD mice, which may be attributed to its effective role in the inhibition of apoptosis and autophagy enhancement.

### 2.5. GM2 Prevents TH Loss

**Figure 5:**
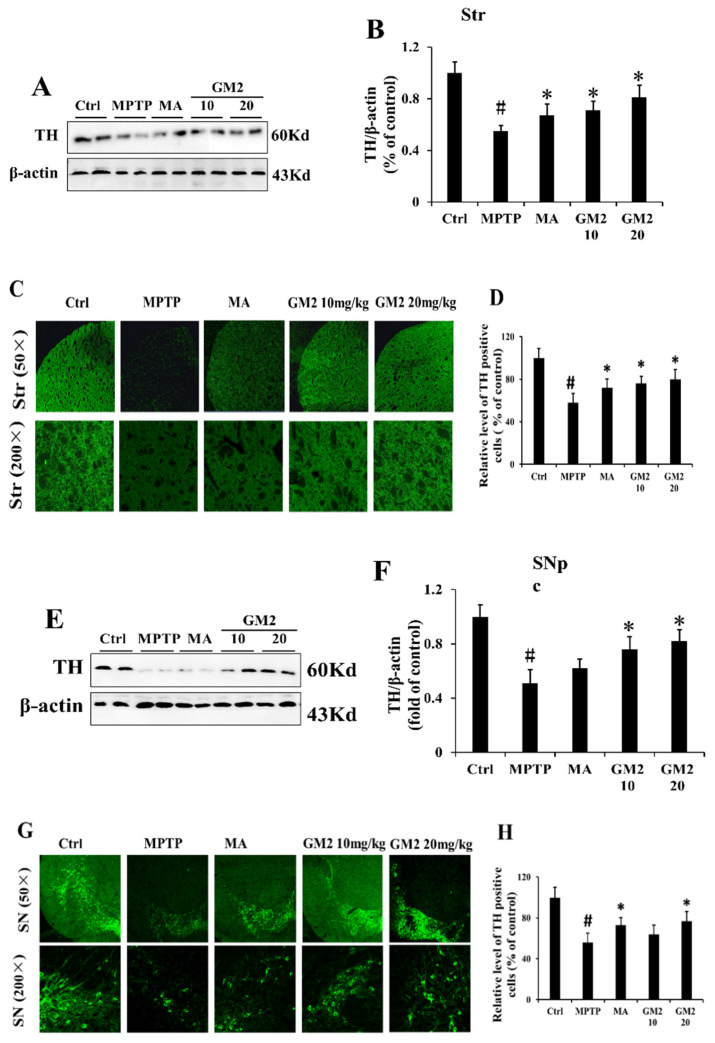
GM2 prevented TH loss in the striatum (Str) and substantia nigra (SNpc). The mice had 7 days to adapt to the environment. Except the control group, the mice in other groups received MPTP hydrochloride intraperitoneally at a dose of 25 mg·kg^−1^·day^−1^ from the 8th day to the 14th day. Then, the mice in GM2 and MA were treated intraperitoneally with GM2 or Madopar from the 15th day to the 19th day: (**A**) the original bands of tyrosine hydroxylase (TH) in Str, (**B**) quantification of TH in Str, (**C**) TH immunostaining in Str, (**D**) the relative level of TH-positive cells in Str, (**E**) the original bands of TH in SNpc, (**F**) quantification of TH in SNpc, (**G**) TH immunostaining in SNpc, and (**H**) the relative level of TH-positive cells in SNpc. Scale bar = 50/200 μm. ^#^ *p* < 0.05 compared to the results in the control group; * *p* < 0.05 compared to the MPTP group. The values are given as mean ± SEM (*n* = 3). Ctrl: the untreated mice; M: the mice treated with MPTP only; GM2: 10 mg·kg^−1^ or 20 mg·kg^−1^; MPTP: 20 mg·kg^−1^.

We found that GM2 enhanced cell viability, inhibited apoptosis, and promoted autophagy in MPP^+^-induced PC12 cells. Our previous study reported that GM2 could ameliorate behavioral deficits and could enhance the level of DA in Parkinsonism mice. Additionally, GM2 enhanced the expression of tyrosine hydroxylase (TH) in striatum by Western blot. In this study, we further assessed the neuroprotective effects of GM2 in MPTP-induced PD mice. We applied Western blot and immunofluorescence to analyze TH in both striatum and substantia nigra. As shown in Figure 5A–D, MPTP induced a reduction in the protein expression of TH and a decrease in TH-positive neurons in the striatum; however, treatment with GM2 markedly prevented TH loss, consistent with previous reports [23]. Similar results were observed in the substantia nigra, showing that GM2 protected dopaminergic neurons from MPTP-induced apoptosis through enhancement of TH levels (Figure 5E–H). Madopar is widely used as a “gold standard” in PD treatment by replenishing striatal DA after oral administration of the DA precursor levodopa. However, in our study, the expression of TH did not improve significantly, especially in the substantia nigra.

**Figure 6:**
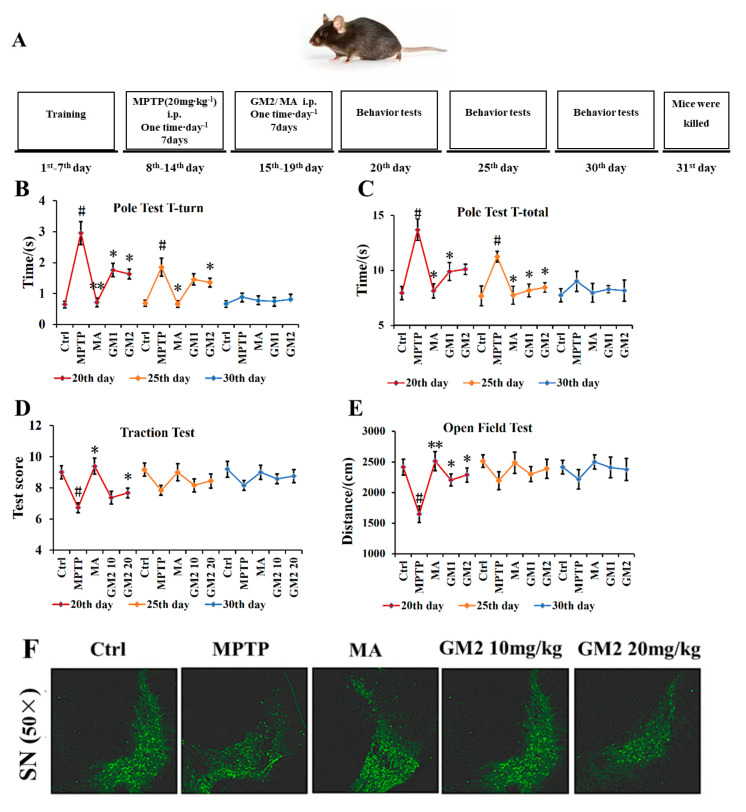
Long term effects of GM2 in MPTP-treated mice: for the long-term experiment, a behavior test was conducted on the mice on the 20th day, 25th day, and 30th day after GM2 treatment: (**A**) the design of the experiment, the T-turn (**B**) and T-total (**C**) of the pole test, (**D**) the traction performance, (**E**) the distance of an open-field performance, and (**F**) TH immunofluorescence of the nigral dopaminergic neurons after GM2 treatment on the 30th day. Scale bar = 50 μm. Samples were taken from mice at 15 days after the last GM2 treatment. ^#^ *p* < 0.05 compared with the control group; * *p* < 0.05 compared with MPTP group, ** *p* < 0.01 compared with MPTP group. A significant difference between MA and GM2 was found (*p* < 0.05). The values are given as mean ± SEM (*n* = 5).

These beneficial effects against MPTP-induced behavioral disorders were prolonged up to 10 days, and the dopaminergic neuron loss was extended up to 15 days after the last GM2 treatment (Figure 6A–F). When treated with Madopar, a large amount of dopamine was added in time, which can lead to a transient state of arousal in mice compared with in the control group.

### 2.6. GM2 Enhanced Autophagy in PD Mice

**Figure 7:**
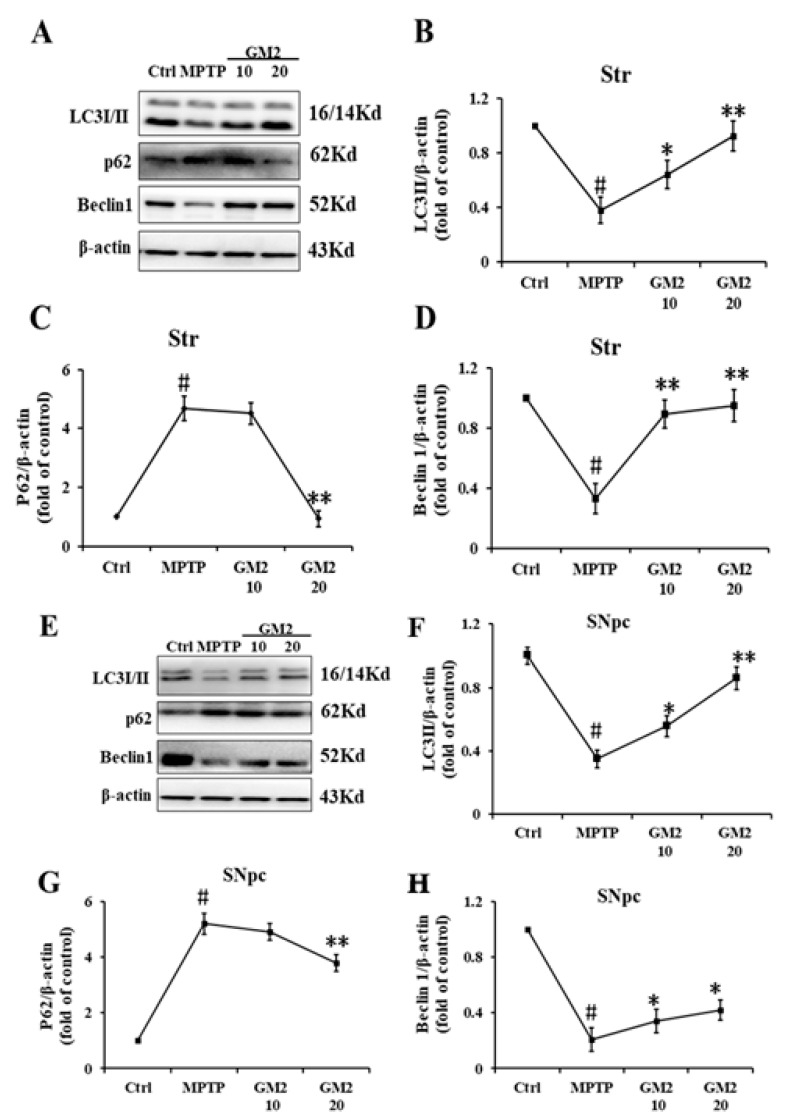
GM2 enhanced autophagy in Parkinson’s disease (PD) mice. The mice had 7 days to adapt to the environment. Except the control group, the mice in the other groups received MPTP hydrochloride intraperitoneally at a dose of 25 mg·kg^−1^·day^−1^ from the 8th day to the 14th day. Then, the mice in GM2 and MA were treated intraperitoneally with GM2 or Madopar from the 15th day to the 19th day: (**A**) the original bands of LC3, p62, and Beclin1 in Str; (**B**) quantification of LC3 in Str; (**C**) quantification of p62 in Str; (**D**) quantification of Beclin1 in Str; (**E**) the original bands of LC3, p62, and Beclin1 in SNpc; (**F**) the ratio of LC3 in SNpc; (**G**) quantification of p62 in Str; and (**H**) quantification of Beclin1 in SNpc. ^#^ *p* < 0.05 versus the control group; * *p* < 0.05 versus MPTP group, ** *p* < 0.01 versus MPTP group. The values are given as mean ± SEM (*n* = 3).

We observed severe apoptosis in the striatum and substantia nigra (SNpc) in PD mice, and GM2 administration reversed this trend. Therefore, we further investigated whether GM2 modulates autophagy in the presence of MPTP. As shown in Figure 7A–D, the levels of Beclin1 and the LC3II/LC3I ratio were significantly downregulated and the level of p62 was elevated following treatment with MPTP, which was largely blocked by the application of GM2. Moreover, we analyzed Beclin1, LC3, and p62 in the SNpc. The results showed that, as well as in the striatum, GM2 greatly prevented MPTP-induced autophagy inhibition (Figure 7E–H). These results suggest that GM2 could enhance autophagy to inhibit apoptosis.

## 3. Discussion

The neurotoxin MPTP is a well-known mitochondrial complex I inhibitor via combining organic cation transporter 3 or the extra neuronal monoamine transporter, which can pass through the blood–brain barrier and transform into its toxic derivative 1-methyl-4-phenylpyridinium (MPP^+^) by monoamine oxidase-B (MAO-B) [34]. MPP^+^ is released from astrocytes and transported into dopaminergic neurons, disrupting mitochondrial respiratory function, compromising ATP synthesis, and inducing excessive intracellular ROS that finally results in dopaminergic neuronal apoptosis. Mitochondria plays an important role in apoptosis through the release of cytochrome c into the cytosol. Accumulation of cytochrome c forms an apoptosome complex with pro-caspase-9 and Apf, leading to caspase-9 activation and further stimulation of the caspase cascade [29,35]. Furthermore, cytosolic cytochrome c interacts with the Bcl-2 family proteins, activating the pro-apoptotic Bax proteins and exacerbating apoptosis [36]. In this study, MPP+ destroyed MMP, attenuated the ratio of Bcl-2/Bax, and damaged the antioxidant capacity in PC12 cells; however, GM2 prevented this trend, implying that GM2 could enhancing mitochondrial function to retard the apoptosis induced by MPP+.

Apoptosis and autophagy are basic cellular processes that are essential for the maintenance of neuronal homeostasis under physiological conditions. Dysfunction of these processes has been reported in various neurodegenerative diseases, including PD [37]. The beneficial roles of autophagy in the nervous system are primarily associated with maintenance of the normal balance between the formation and degradation of cellular proteins [38]. A number of studies have suggested that the attenuation of autophagy or incomplete progress in degradation pathways are important initiators of PD [38,39]. Interesting evidence has shown that the induction of autophagy with trehalose results in improvements in the molecular traits of PD [40]. Beclin1, p62, and LC3 are key indicators for the detection of autophagy. Upon autophagy stimulation, Beclin1 is released from Bcl-2 at the endoplasmic reticulum, triggering Atg5 multimeric complex formation to further incorporate with LC3 II, which is lipidated, facilitating its incorporation into the nascent phagophore membrane. LC3 Ⅱ is involved in the extension of the autophagic lysosomal membranes and then binds to p62 localized in the cytoplasm to form a complex, which is eventually degraded in lysosomes [41]. In this study, we found that GM2 enhanced autophagy by improving the levels of the LC3II ratio and Beclin1 and by decreasing the level of p62 both in MPP^+^-treated PC12 cells and MPTP-induced Parkinsonism mice. These results revealed that GM2 might play an important role in improving mitochondrial function and autophagy in PD models.

Our previous study reported that the glucuronomannan oligosaccharide GM2 from *Saccharina japonica* could ameliorate behavioral deficits and increased the protein level of TH in the striatum of Parkinsonism mice [23]. TH, the rate-limiting enzyme in the synthesis of DA, is often used as a marker for the integrity of dopaminergic neurons [42,43]. Parkinson’s disease (PD) is characterized by marked degeneration of dopaminergic substantia nigra (SN) neurons. In this study, the improvement in TH expression after GM2 treatment both in the striatum and substantia nigra by Western blot and immunofluorescence (Figure 6) further verified that GM2 played a neuroprotective role in PD mice.

## 4. Materials and Methods

### 4.1. Materials

Madopar (MA) was obtained from Shanghai Roche Pharmaceuticals Ltd (Shanghai, China); MPTP was purchased from Sigma-Aldrich (Milwaukee, WI, USA). Rabbit antibodies to TH (#ab112), Bax (#ab32503), Bcl-2 (#ab182858), Cyt c (#ab133504), LC3 (#ab48394), p62 (#ab109012), Beclin1 (#ab207612), and β-actin (#ab8226) were obtained from Abcam, Cambridge, UK.

Adult male C57BL/6 mice (8–10 weeks, weighing 25–30 g) were kept with free access to food and tap water with constant temperature (22 ± 2) °C and relative humidity (60 ± 10)%.

**Figure 8:**
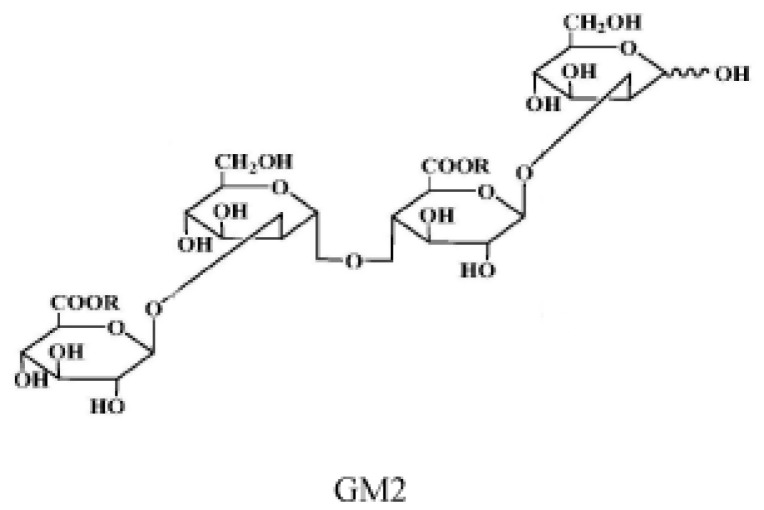
The structure of GM2.

For the preparation of GM2, please refer to a previous study (Figure 8) (see the Supplementary Materials) [23].

### 4.2. Cell Culture

PC12 cells, a rat adrenal pheochromocytoma cell line (between 15–25 passage) obtained from iCell Bioscience Inc, Shanghai (Shanghai, China), were cultured in T-25 flasks at 37 °C in 95% air/5% CO_2_ and maintained in Dulbecco’s modified eagle medium (DMEM) supplemented with 10% heat-inactivated horse serum, 5% fetal bovine serum (FBS), 100 U·mL^−1^ penicillin, and 100 μg·mL^−1^ streptomycin. Neuronal differentiation of PC12 cells was induced by (nerve growth factor) NGF (100 ng·mL^−1^; R&D Systems, Minneapolis, MN, USA) in DMEM containing 1% FBS every other day for 3 days.

Cells were treated with different concentrations of GM2 or MPP^+^ for 24 h to detect the cytotoxicity. PC12 cells were treated with GM2 for 12 h and, after, were exposed to 2 mmol·L^−1^ MPP^+^ for 24 h. The control cells were treated with media containing the same volume of PBS only. The cell viability was measured by the CCK-8 regent and LDH leakage.

### 4.3. Annexin V/Propidium Iodide (PI) Staining

The cells were treated as above. After trypsinization, the cells were suspended in annexin-binding buffer. The cell suspension (1 × 10^5^ cells∙mL^−1^) was incubated with fluorescein isothiocyanate (FITC)-conjugated anti-annexin V and propidium iodide (PI) for 15 min in the dark. The fluorescence spectra of annexin V and PI were detected using 527/32 nm and 586/42 nm filters, respectively.

### 4.4. Measurement of MMP

MMP was quantified using a JC-1 fluorescent probe (Sigma-Aldrich, St. Louis, MO, USA). PC12 cells were treated as described above. PC12 cells were incubated with 1 mL of the 10 µg•mL^−1^ JC-1 probe for 15 min at 37 °C in the dark. The JC-1 monomer (low membrane potential) was measured at the excitation wavelengths of 514 nm. The JC-1 aggregates (high membrane potential) were measured at the excitation wavelengths of 585 nm. The colored images were obtained using a laser scanning confocal microscope (Olympus Fluoview FV1000, Tokyo, Janpan). The quantification of fluorescence intensity was measure with a microplate reader (Tecan Infinite M1000 PRO, Männedorf, Switzerland)

### 4.5. Measurement of ROS

Intracellular ROS levels were analyzed using a ROS assay kit (Nanjing Jiancheng Bioengineering Institute, Nanjing, China). PC12 cells were seeded into 6-well plates at 2 × 10^5^ cells·well^−1^. Cells were treated as described above. After rinsing three times, cells were incubated with 10 µmol·L^−1^ dichlorofluorescein diacetate (DCFH-DA) at 37 °C for 40 min. Images were obtained using a laser scanning confocal microscope (Olympus Fluoview FV1000). Quantification of the fluorescence intensity was measured with a microplate reader (Tecan Infinite M1000 PRO, Männedorf, Switzerland).

### 4.6. Antioxidant Systems Assay

Cells were seeded into 6-well plates at 2 × 10^5^ cells·well^−1^. Cells were homogenized in 0.4 mL of 0.2% Triton X-100 in PBS and centrifuged at 12,000 rpm·min^−1^ for 3 min, and supernatants were collected for use in a subsequent assay. SOD and GSH were measured using commercial assay kits (Nanjing Jiancheng Bioengineering Institute, Nanjing, China) according to the manufacturer’s instructions. Total protein concentration was detected using the bicinchoninic acid (BCA) kit.

### 4.7. Determination of Caspase-3 and Caspase-9 Activity

The activities of caspase-3 and caspase-9 were measured using a colorimetric caspase-3/-9 assay kit according to the manufacturer’s protocol (Beyotime Biotechnology, China). The reaction mixture contained 30 μL of cell lysate and 10 μL of the caspase-3 or caspase-9 substrate (Ac-DEVD-pNA or Ac-LEHD-pNA, final concentration 200 μmol·L^−1^) in the assay buffer. The mixtures were incubated at 37 °C for 3 h, and the absorbance was read at 405 nm.

### 4.8. Drug Administration

This study was approved by the Animal Care and Ethics Committee of the Affiliated Hospital of Qingdao University in compliance with the Principles of Laboratory Animal Care developed by the National society for Medical Research. The Laboratory animal permit was SYXK (Lu) 2015 0003. The date of approval was 27 Feburary 2019.

The mice had 7 days to adapt to the environment and were randomly assigned into five groups (*n* = 12): (1) control, (2) MPTP only, (3) MPTP + Madopar (70 mg·kg^−1^), (4) MPTP + GM2 (10 mg·kg^−1^), and (5) MPTP + GM2 (20 mg·kg^−1^). The mice in groups (2–5) received MPTP hydrochloride intraperitoneally at a dose of 25 mg·kg^−1^·day^−1^ from the 8th day to the 14th day. Group (1) received the same volume of saline. Then, groups (3–5) were treated intraperitoneally with GM2 or Madopar from the 15th day to the 19th day.

For the long-term experiment, a behavior test was conducted on the mice (*n* = 5) on the 20th day, 25th day, and 30th day after GM2 treatment (Figure 6A).

### 4.9. Western Blot Analysis

After GM2 treatment, the striatum in 7 mice were collected and lysed in lysis buffer on ice for 30 min followed by centrifugation at 12,000 rpm for 10 min. Protein concentrations were determined using a BCA assay kit, and equal amounts of protein were separated by 10–15% SDS–PAGE gels. Thereafter, the proteins were transferred to a poly(vinylidene difluoride) (PVDF) membrane and blocked with 5% bovine serum albumin for 2 h. Membranes were immunoblotted with specific antibodies (BAX, ab32503; Bcl-2, ab182858; LC3, ab192890; P62, 109012; Beclin1, ab210498; and TH, ab112) overnight at 4 °C and then applied to appropriate horseradish peroxidase-conjugated anti-mouse or anti-rabbit IgG secondary antibodies (1:2000) for 1 h. Signals were detected using an enhanced chemiluminescence.

### 4.10. Tissue Collection and Preparation: Immunostaining

Five mice from each group were anesthetized and perfused with an intracardial infusion of saline, followed by 4% paraformaldehyde. Brains were subsequently post-fixed in the same fixative for 48 h and then dehydrated in 20% and 30% sucrose, successively, at 4 °C. Brains were sectioned at a thickness of 16 μm on a sliding microtome for immunofluorescence (Leica CM1520, Solms, Germany). Sections were incubated with 0.2% Triton X-100 for 15 min at 25 ± 5 °C and blocked in 5% goat serum for 30 min at 37 °C. Then, the sections were stained with a primary antibody for against tyrosine hydroxylase (TH) (1:100) overnight at 4 °C. After washing three times with PBS, sections were incubated with Alexa Fluor ^®®^ 488 goat anti-rabbit IgG (H+L; Eugene, OR, USA; 1:200) for 1 h. Positive neurons were detected in five to six sections using a scanning confocal microscope (Olympus Fluoview FV1000). Fluorescence intensity was quantified using Image J software. The average fluorescence intensity was calculated from 6–8 distributed areas in the sample images.

### 4.11. Statistical Analysis

Each experiment was performed independently at least three times, and the data are presented as mean ± SEM for the indicated number of separate experiments; 17.0 SPSS software (SPSS Inc., Chicago, IL, USA) was applied to analyze data, and one-way ANOVA was used to compare the mean differences among the groups. *p*-values < 0.05 were considered significant (^#^ *p* < 0.05 compared with the control group; * *p* < 0.05 and ** *p* < 0.01 compared with the MPTP group).

## 5. Conclusions

In this study, we applied GM2 with the single and definite structure to investigate potential neuroprotective effects in a PD model. The results demonstrated that treatment with GM2 reduced apoptosis and enhanced mitochondrial function in PC12 cells. Furthermore, GM2 prevented dopaminergic neuron loss in PD mice through an enhancement of autophagy. These results suggest that GM2 might represent a promising agent to fight PD through enhancement of mitochondrial function and autophagy.

## Supplementary Materials

The following are available online at https://www.mdpi.com/1660-3397/19/2/58/s1, Figure S1: The structure of GM1, GM2 and GM3. Table S1: The chemical shifts of GM2.
