# Supplementary material for: Glucuronomannan GM2 from Saccharina japonica Enhanced Mitochondrial Function and Autophagy in a Parkinson’s Model
Source: Mar Drugs. 2021 Jan 25;19(2):58. doi: 10.3390/md19020058 (PMC7912055; doi:10.3390/md19020058)
Supplement: Supplementary file 1 [file marinedrugs-19-00058-s001.pdf]

Supplementary Materials

Glucuronomannan GM2 from *Saccharina japonica* Enhanced Mitochondrial Function and Autophagy in a Parkinson's Model

Yingjuan Liu [1,2,3], Weihua Jin [4], Zhenzhen Deng [1,3], Quanbin Zhang [1,3] and Jing Wang [1,3,*]

1 Key Laboratory of Experimental Marine Biology, Center for Ocean Mega-Science, Institute of Oceanology, Chinese Academy of Sciences, Qingdao 266071, China; liuyjmiao@163.com (Y.L.); dengzhenzhen16@mails.ucas.ac.cn (Z.D.); qbzhang@qdio.ac.cn (Q.Z)
2 School of Basic Medicine, Qingdao University, Qingdao 266071, China
3 Laboratory for Marine Biology and Biotechnology, Qingdao National Laboratory for Marine Science and Technology, Qingdao 266071, China
4 College of Biotechnology and Bioengineering, Zhejiang University of Technology, Hangzhou 310014, China; jinweihua@zjut.edu.cn
* Correspondence: jingwang@qdio.ac.cn; Tel./Fax: +86-532-8289-8703

Preparation of Glucuronomannan Oligosaccharides (GM2)

Glucuronomannan oligosaccharide (GM2) was prepared according to a protocol reported in a previous study [1]. Briefly, crude fucoidan from *Saccharina japonica* was hydrolyzed by oxidative degradation by combination with hydrogen peroxide and ascorbic acid (30 mM). Then, it was ultrafiltrated, concentrated and precipitated by 75% ethanol before further precipitation with 95% ethanol. Finally, the degraded polysaccharides underwent anion exchange chromatography on a DEAE-Bio Gel Agarose FF gel (12 cm × 70 cm) with elution by 0.5 M, 1 M and 2 M NaCl. F0.5 was dissolved in 4% dilute sulfuric acid to a final concentration of 60 mg/mL, degraded at 100 °C for 5 h, cooled to room temperature, and then neutralized with barium hydroxide. After centrifugation, the supernatant was concentrated and fractionated on an activated carbon column chromatography. Fraction Y1 was obtained from eluent with water and Fraction Y2 was from eluent with 50% ethanol. Y2 was further separated on a Bio-Gel P-4 Gel column eluted with 0.5 M NH_4HCO_3 solution.

After degradation by sulfuric acid, we obtained GM1 (disaccharide), GM2 (tetraose), GM3 (hexose), GM4 (octaose) and some monosaccharaides. The purity of GM2 was identified before it was applied to this experiment. The purities of GM2 were more than 90%.

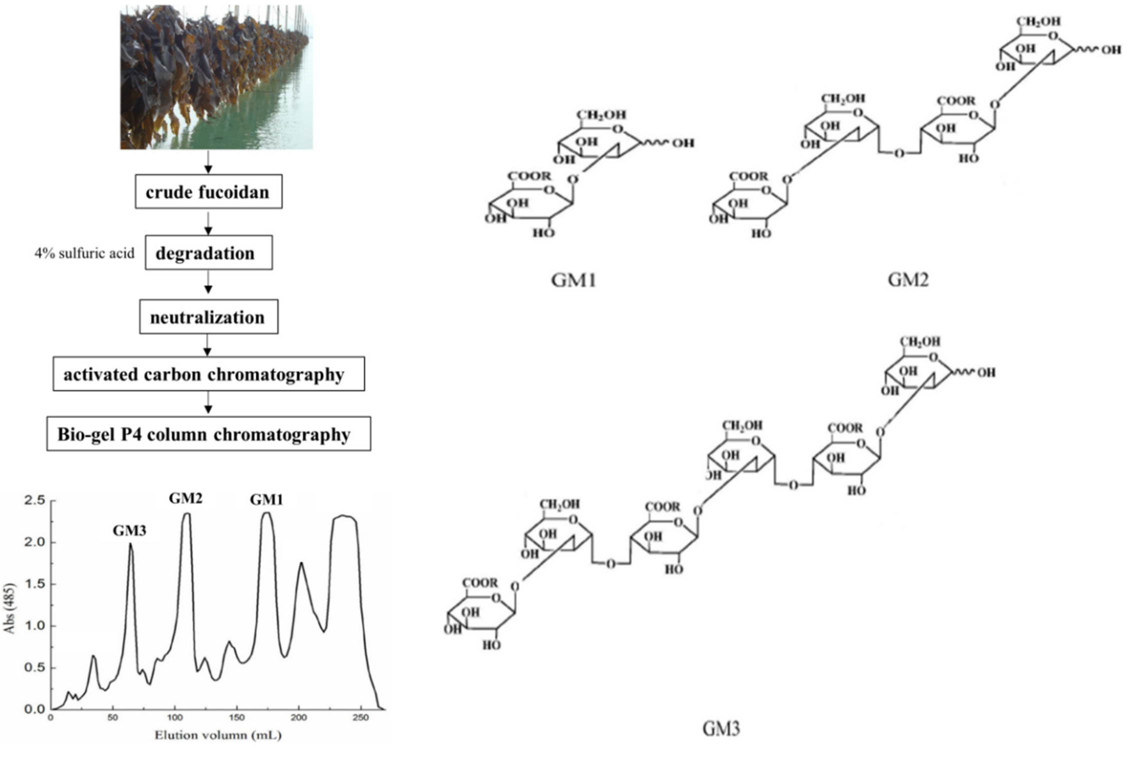

Figure S1. The structure of GM1, GM2 and GM3. (The structure of GM2 was shown in Figure 8 in submitted manuscript).

Table S1. The chemical shifts of GM2 [2].

Samples	Residues	C1	C2	C3	C4	C5	C6	H1	H2	H3	H4	H5	H6
GM2	→2)-α-D-Manp-OH	92.4	78.6	69.8	67.4	72.7	60.9	5.1[illegible]	3.9[illegible]	3.7[illegible]	3.5[illegible]	3.5[illegible]	3.6–3.7
	→4)-β-D-GlcAp-(1→	101.9	73.0	76.7	77.2	76.4	175.3	4.3[illegible]	3.2[illegible]	3.5[illegible]	3.6[illegible]	3.6[illegible]	–
	→2)-α-D-Manp-(1→	98.8	77.9	69.9	66.8	72.9	60.4	5.2[illegible]	4.0[illegible]	3.7	3.5[illegible]	3.5[illegible]	3.6–3.7
	β-D-GlcAp-(1→	101.8	73.1	75.6	72.2	76.2	176.2	4.3[illegible]	3.2[illegible]	3.3[illegible]	3.6[illegible]	3.6[illegible]	–

Reference:

1. Liu, Y.; Jin, W.; Deng, Z.; Wang, J.; Zhang, Q., Preparation and neuroprotective activity of glucuronomannan oligosaccharides in an MPTP-induced Parkinson's model. *Mar Drugs* **2020,** 18, (9).
2. Jin, W.; He, X.; Zhu, J.; Fang, Q.; Wei, B.; Sun, J.; Zhang, W.; Zhang, Z.; Zhang, F.; Linhardt, R. J.; Wang, H.; Zhong, W., Inhibition of glucuronomannan hexamer on the proliferation of lung cancer through binding with immunoglobulin G. *Carbohydr Polym* **2020,** 248, 116785.
